# Microscopic and metabolic investigations disclose the factors that lead to skin cracking in chili-type pepper fruit varieties

**DOI:** 10.1093/hr/uhad036

**Published:** 2023-02-28

**Authors:** Ofir Marinov, Gal Nomberg, Sutanni Sarkar, Gulab Chand Arya, Eldad Karavani, Einat Zelinger, Ekaterina Manasherova, Hagai Cohen

**Affiliations:** Department of Vegetable and Field Crops, Institute of Plant Sciences, Agricultural Research Organization (ARO), Volcani Institute, Rishon LeZion 7505101, Israel; Department of Plant Pathology and Microbiology, The Robert H. Smith Faculty of Agriculture, Food and Environment, The Hebrew University of Jerusalem, Rehovot 7610001, Israel; Department of Vegetable and Field Crops, Institute of Plant Sciences, Agricultural Research Organization (ARO), Volcani Institute, Rishon LeZion 7505101, Israel; Department of Plant Pathology and Microbiology, The Robert H. Smith Faculty of Agriculture, Food and Environment, The Hebrew University of Jerusalem, Rehovot 7610001, Israel; Department of Vegetable and Field Crops, Institute of Plant Sciences, Agricultural Research Organization (ARO), Volcani Institute, Rishon LeZion 7505101, Israel; Department of Plant Pathology and Microbiology, The Robert H. Smith Faculty of Agriculture, Food and Environment, The Hebrew University of Jerusalem, Rehovot 7610001, Israel; Department of Vegetable and Field Crops, Institute of Plant Sciences, Agricultural Research Organization (ARO), Volcani Institute, Rishon LeZion 7505101, Israel; Department of Vegetable and Field Crops, Institute of Plant Sciences, Agricultural Research Organization (ARO), Volcani Institute, Rishon LeZion 7505101, Israel; Center for Scientific Imaging (CSI), The Robert H. Smith Faculty of Agriculture, Food and Environment, The Hebrew University of Jerusalem, Rehovot 7610001, Israel; Department of Vegetable and Field Crops, Institute of Plant Sciences, Agricultural Research Organization (ARO), Volcani Institute, Rishon LeZion 7505101, Israel; Department of Vegetable and Field Crops, Institute of Plant Sciences, Agricultural Research Organization (ARO), Volcani Institute, Rishon LeZion 7505101, Israel

## Abstract

The hydrophobic cuticle encasing the fruit skin surface plays critical roles during fruit development and post-harvest. Skin failure often results in the fruit surface cracking and forming a wound-periderm tissue made of suberin and lignin. The factors that make the fruit skin susceptible to cracking have yet to be fully understood. Herein, we investigated two varieties of chili peppers (*Capsicum annuum* L.), Numex Garnet, whose fruit has intact skin, and Vezena Slatka, whose fruit has cracked skin. Microscopical observations, gas chromatography–mass spectrometry, biochemical and gene expression assays revealed that Vezena Slatka fruit form a thicker cuticle with greater levels of cutin monomers and hydroxycinnamic acids, and highly express key cutin-related genes. The skin of these fruit also had a lower epidermal cell density due to cells with very large perimeters, and highly express genes involved in epidermal cell differentiation. We demonstrate that skin cracking in the Vezena Slatka fruit is accompanied by a spatial accumulation of lignin-like polyphenolic compounds, without the formation of a typical wound-periderm tissues made of suberized cells. Lastly, we establish that skin cracking in chili-type pepper significantly affects fruit quality during post-harvest storage in a temperature-dependent manner. In conclusion, our data highlight cuticle thickness and epidermal cell density as two critical factors determining fruit skin susceptibility to cracking in chili-type pepper fruit.

## Introduction

As all aboveground tissues of plants, the outermost epidermal cell layer in the skin of fleshy fruit is encased by a hydrophobic cuticle. Compared to the cuticles that cover the surfaces of vegetative tissues, the cuticle that coats the fruit skin is of several orders thicker [1]. The predominant component that builds the cuticle is the cutin polymer - a mixture of C16 and C18 ω-hydroxylated fatty acids that are cross-linked via ester bonds and attached to a glycerol backbone. Previous evidence showed that apart from these components, cutin can also contain dicarboxylic acids, alkanoic acids and phenylpropanoid derivatives [2]. In some fruit species, *e.g.* tomato and persimmon, the cuticles were shown to accumulate the cell wall polysaccharides pectin and cellulose [3–5]. The cuticle also contains epicuticular waxes deposited above the cuticle in the form of crystals, as well as intracuticular waxes that are immersed within the cutin matrix. Waxes are made from very-long-chain fatty acids (VLCFAs) along with their byproducts from the biochemical classes of alcohols, esters, aldehydes, ketones, triterpenoids and sterols [6–8].

The cuticle plays pivotal roles during fruit development. It regulates the transport of water and gases into and out of the fruit, protects the fruit from invading pathogens, and shields the internal fleshy tissues from damaging UV radiation [9]. Consequently, fruit skin failure has detrimental consequences for fruit development and physiology. It also reduces the fruit’s quality and marketability, owing to the damaged appearance [10]. Recently, skin cracking in cucumber fruit was shown to significantly affect fruit performance under various conditions of post-harvest storage [11]. Fruit skin failure typically occurs when the fruit skin cannot withstand the tension forces arising due to the ongoing strain applied on the outermost epidermal layer and skin tissues during fruit growth and expansion. There are a range of skin failure phenotypes, from skin browning and spotting to shriveling, russeting, splitting, reticulating and cracking [12]. The later phenotype can assume the form of micro-cracking, where fissures are confined to the cuticle, or that of macro-cracking, where fissures penetrate the cuticle and the inner parenchymal cell layers underneath the cuticle.

Once this mechanical damage occurs, some species of fleshy fruit are capable of restoring skin surface integrity by forming a secondary wound-periderm tissue–a specialized corky coating above the skin made of the aromatic polymers suberin and lignin [13, 14]. Suberin is a complex polymer built from two domains: a poly(aliphatic) domain consisting of >C20 VLCFA derivatives such as ω-hydroxyacids, dihydroxyacids and fatty alcohols; and a poly(phenolic) domain made of glycerol-based alkyl ferulates and phenylpropanoid-derived hydroxycinnamic acids [15, 16]. The lignin polymer is derived from three phenolic hydroxycinnamyl monomers, namely, *p*-coumaryl, coniferyl and sinapyl alcohols [17]. The formation of a wound-periderm is vital for the continuous development of the injured fruit: it preserves skin integrity and elasticity [18–20]; maintains the turgor pressure of cells lying below the skin and partially restores fruit skin permeability to water losses [21–24]; and helps prevent additional mechanical injury [25].

Despite the agricultural significance of skin cracking in terms of fruit quality, both pre- and post-harvest, the chain of events and factors leading to the cracking of fruit skin are poorly understood. Herein, we aimed at investigating the factors underlying skin cracking in varieties of pepper (*Capsicum annuum* L.) with an elongated chili-type fruit structure, an important crop belonging to the *Solanaceae* family. This included the Numex Garnet variety, a paprika-type fruit characterized by high extractable color, low pungency, and a smooth intact skin that does not crack during fruit development [26]. The second variety is Vezena Slatka, a chili-type fruit whose skin surface is severely cracked, often exhibiting a corky-like appearance [27].

## Results

### The skin of Vezena Slatka fruit undergoes severe cracking during development

**Figure 1:**
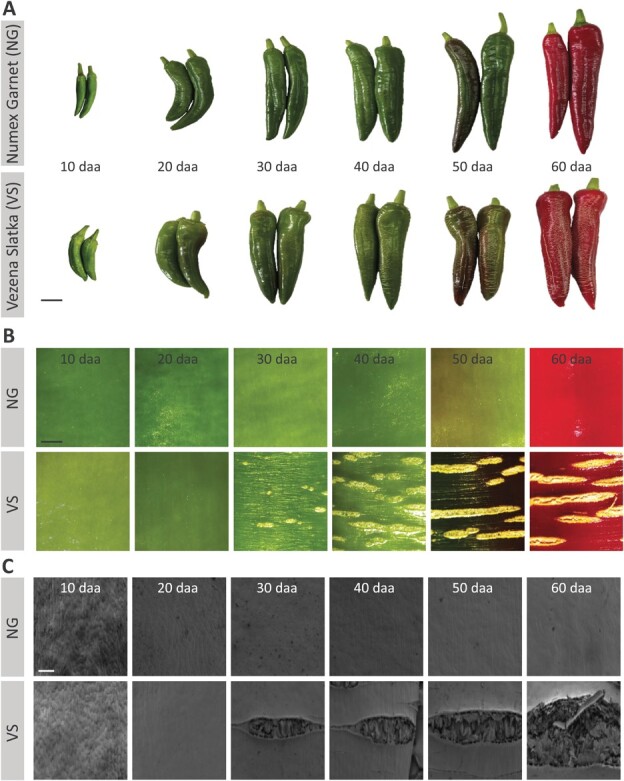
The skin of Vezena Slatka fruit undergoes severe cracking during development**. (A)** The fruit phenotypes, **(B)** light microscopy images of the fruit skin surfaces, and **(C)** scanning electron microscopy (SEM) images of fruit skin surfaces, of smooth-skin Numex Garnet (NG) and cracked-skin Vezena Slatka (VS) pepper at 10, 20, 30, 40, 50 and 60 days after anthesis (daa). Scale bars in (A) = 2 cm, in (B) = 5 mm, and in (C) = 300 μm.

We characterized the fruit phenotypes of both varieties at 10, 20, 30, 40, 50, and 60 days after anthesis (daa), that displayed similar developmental, ripening and maturity stages. Their skin turned red at 50 daa and became fully mature and red-colored at 60 daa, at which point both fruit reached a similar size (Fig. 1A). Light and scanning electron microscopy (SEM) of the fruits’ skin surface demonstrated that the skin of Numex Garnet fruit remained intact throughout fruit development and maturity (Fig. 1B-C), whereas that of Vezena Slatka fruit displayed severe horizontal cracks that interrupted the fruit surface perpendicularly to the fruit longitudinal axis (Fig. 1B-C). At 30 daa, we noticed the first signs of cracking, which protracted and elongated over the course of development, with the fully mature fruit almost completely covered by thick cracks (Fig. 1B-C). SEM images of the cracked areas revealed large openings in the fruit skin where ruptured epidermal cells coated by cuticle protrude above the fruit skin surface (Supplementary Fig. S1).

### The cuticle of Vezena Slatka fruit accumulates higher levels of cutin monomers and hydroxycinnamic acids

**Figure 2:**
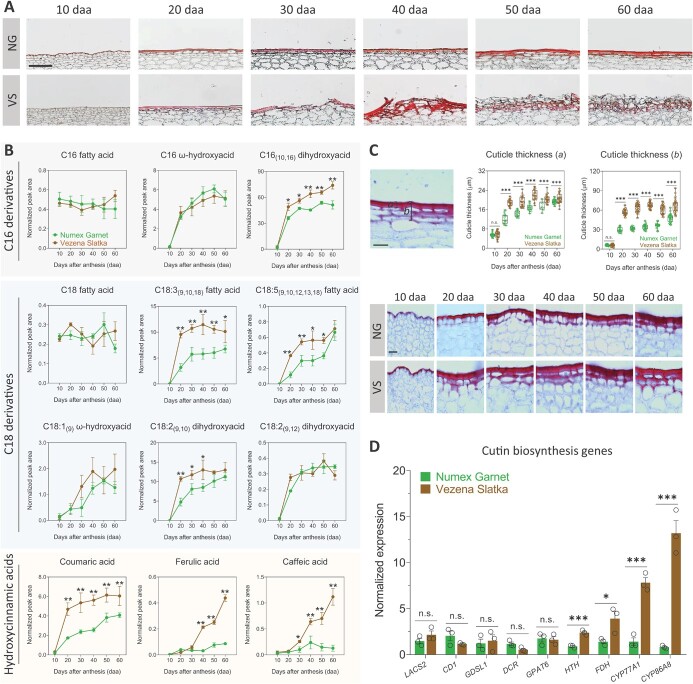
Vezena Slatka fruit form a thicker cuticle with greater levels of cutin monomers and hydroxycinnamic acids, and highly express key cutin-related genes. **(A)** Skin histological cross-sections of smooth-skin Numex Garnet (NG) and cracked-skin Vezena Slatka (VS) fruit at 10, 20, 30, 40, 50 and 60 days after anthesis (daa). Sections were histochemically stained for the skin cuticle layer by Sudan IV. **(B)** Metabolite profiling of cutin monomers in skin of both fruit during development. Line graphs represent the levels of individual metabolites in de-lipidated and trans-esterified skin tissues, following gas chromatography–mass spectrometry (GC–MS) profiling where y-axes represent the relative peak areas following normalization to a C32 alkane internal standard. **(C)** Measurements of cuticle thickness in skin histological sections stained with Sudan IV. The cuticular membrane deposited outside the epidermal cells (*a*) and the entire width of the cuticular membrane that covers epidermal and/or sub-epidermal cells (*b*) were measured. **(D)** Transcript expression levels of key genes involved in cuticle biosynthesis in skin tissues of 50 daa fruit from both types. Gene expression levels were measured by quantitative real-time PCR (qRT-PCR) where y-axis represents the normalized expression of each gene following normalization to the two *Capsicum annuum* reference genes *Polyubiquitin-like* (CA00g79660) and *UBI-3* (Capana04g002411). Full gene names appear in the main text and in Materials and Methods. The data in (B) and (D) represent means ± SE of three biological replicates (generated from a pool of skin tissue from four different fruit), and in (C) means ± SE of 30 biological replicates (generated from individual fruit). The significance (B), (C) and (D) was calculated according to a *t*-test of *p*-values: ^*^p < 0.05; ^**^p < 0.01; ^***^p < 0.001. n.s. = non-significant. Scale bars in (A) = 100 μm, and in (C) = 50 μm.

Skin cracking was previously associated with fruit cuticle characteristics. We therefore sought to determine any structural and biochemical differences between the skin cuticle of Numex Garnet and Vezena Slatka fruit. First, we histologically-sectioned skin samples from both fruit at all six investigated developmental stages, which were stained for the cuticle layer using the histochemical dye Sudan IV. Numex Garnet fruit skin sections demonstrated an intact cuticle with no visible skin cracking. The cuticle layer appeared to expand and widen throughout the development and maturity of the fruit (Fig. 2A). Nonetheless, fruit of the Vezena Slatka exhibited a modified skin anatomy and structure during development. In accord with light and electron microscopy observations, intact epidermis cells coated with an integral cuticle were detected in 10 and 20 daa fruit skin sections. At 30 daa, however, we noticed the initiation of cracking in which the stained cuticle and the epidermal cell layer underneath seem to be broken, resulting in the bulging of these cell layers upward from the fruit’s skin surface (Fig. 2A). From this point onward, the cuticle along the cell layers underneath seem to further disassociate and lose their typical structure (Fig. 2A).

Next, to ascertain the content and composition of the cutin polymer that builds the skin cuticle in both fruit, we chemically-profiled skin tissues via gas chromatography–mass spectrometry (GC–MS)-based protocols [13, 14, 28]. Overall, we were able to positively identify 12 cutin monomers in both fruit (three 16 fatty acid derivatives, six C18 fatty acid derivatives, and three hydroxycinnamic acids) (Fig. 2B). C16 fatty acid and C16 ω-hydroxyacid showed similar accumulation patterns along the development of Numex Garnet and Vezena Slatka fruit, however, C16_(10,16)_ dihydroxyacid - the predominant cutin building block - was significantly higher in Vezena Slatka fruit skin from 20 daa onwards (Fig. 2C). Out of the six C18 fatty acid derivatives identified, C18 fatty acid, C18:1_(9)_ ω-hydroxyacid and C18:2_(9,12)_ dihydroxyacid exhibited comparable levels in skin tissues of both fruit (Fig. 2C). Though, significantly higher levels of fatty acids C18:3_(9,10,18)_ and C18:5_(9,10,12,13,18)_, and the dihydroxyacid C18:2_(9,10)_, were found in the skin of Vezena Slatka fruit in most developmental stages (Fig. 2B). Lastly, the analysis also inferred significantly higher levels of three hydroxycinnamic acids - coumaric, ferulic and caffeic acids - in Vezena Slatka fruit compared to Numex Garnet (Fig. 2C). Markedly, ferulic and caffeic acid buildups in Vezena Slatka fruit first appeared at 30 daa and displayed a constant increase along fruit development, which corresponded to the appearance of cracks in those fruit (Fig. 2B). Unlike these two monomers, coumaric acid was accumulated in skin tissues of both fruit species already at 20 daa, and from this developmental stage until full maturity displayed continual increased levels in skin of both fruit, which were more marked in Vezena Slatka fruit (Fig. 2B). Altogether, the differences in the levels of these individual cutin-associated metabolites resulted in significantly higher loads of cutin contents in Vezena Slatka fruit compared to Numex Garnet from 20 daa until full maturity at 60 daa, ranging from 25% to 58%.

We speculated that the higher synthesis of several key cutin monomers resulting in higher total cutin contents during the development of Vezena Slatka fruit might lead to the deposition of a thicker cuticle. We used skin histological sections stained with Sudan IV to measure the thickness of the cuticular membrane deposited outside the epidermal cells (*a*) and the entire width of the cuticular membrane that covers epidermal and/or sub-epidermal cells (*b*), based on a recent protocol [29] (Fig. 2C). In the Vezena Slatka fruit we measured the cuticle in skin surfaces that were not cracked. At 10 daa, both fruit deposited a similar ~6 μm cuticular membrane *a* without penetrating towards the sub-epidermal cells (Fig. 2C). However, at 20 daa we detected a 1.5- and 1.9-fold thicker cuticular membranes *a* and *b* in Vezena Slatka fruit compared to Numex Garnet (*a* = 19 μm and 12 μm, respectively; *b* = 57 μm and 29 μm, respectively; Fig. 2C). Generally, this trend was observed throughout fruit skin development until full maturity at 60 daa, where Vezena Slatka fruit deposit significant 6% and 40% thicker cuticular membranes *a* and *b*, respectively (*a* = 21 μm and 19 μm, respectively; *b* = 67 μm and 48 μm, respectively; Fig. 2C). Overall, these measurements point at 20 daa as a key developmental stage where major differences in cuticular membrane thickness exist between the two fruit.

Finally, we measured the expression of genes involved in the cutin biosynthesis pathway in skin samples of 50 daa fruit from both varieties via qRT-PCR, expecting to find higher expression in the skin of Vezena Slatka fruit that accumulated more cutin contents. Yet, we found similar expression patterns of several key cutin biosynthesis genes in the skin of both fruit. Among these genes are *LONG-CHAIN ACYL-COA SYNTHETHASE 2* (*LACS2*), which catalyzes the synthesis of $ \omega $-hydroxy fatty acyl-CoA intermediates [30]; *CUTIN DEFICIENT 1* (*CD1*) and *GDSL1*, both of which encode GDSL-motif lipase/hydrolase family proteins involved in cutin monomer polymerization [31, 32]; *DEFECTIVE in CUTICULAR RIDGES* (*DCR*), which encodes a soluble BAHD acyltransferase that incorporates dihydroxyacid C16_(10,16)_ into cutin and plays a role in cuticle organization [33, 34]; and *GLYCEROL-3-PHOSPHATE ACYL TRANSFERASE 6* (*GPAT6*) involved in the synthesis of monoacylglycerols [35, 36] (Fig. 2D). Still, four other key genes were expressed more in Vezena Slatka fruit. Namely, we found 2.8-fold higher expression of *FIDDLEHEAD* (*FDH*) encoding an epidermis-specific 3-KETOACYL-COA SYNTHASE that synthesizes long-chain fatty acids [37]; and 2.7-, 5.5- and 16.4-fold higher expression of *HOTHEAD* (*HTH*) and P450 cytochromes *CYP77A1* and *CYP86A8*, respectively, all of which participate in the synthesis of long-chain ω-hydroxyacids and dihydroxyacids in the cuticle [23, 35, 38, 39] (Fig. 2D). Indeed, the chemical composition of cutin in the skin of Vezena Slatka fruit pointed at the accumulation of the monomers from these biochemical classes.

### The skin of Vezena Slatka fruit has lower epidermal cell density due to cells with larger perimeters, and highly expresses genes involved in epidermal cell differentiation

**Figure 3:**
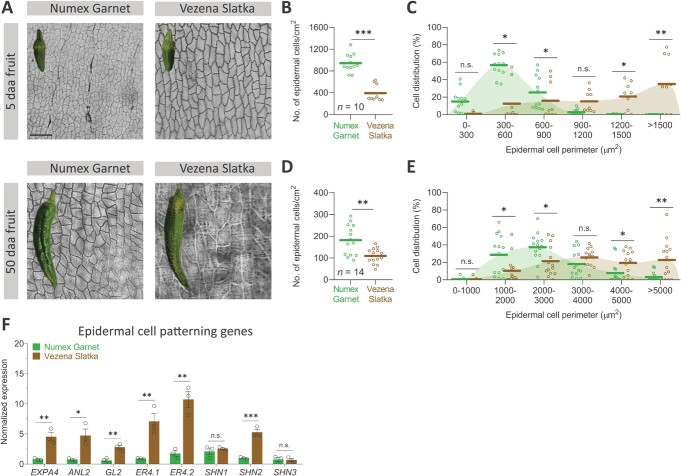
The skin of Vezena Slatka fruit has lower epidermal cell density due to cells with larger perimeters, and highly expresses genes involved in epidermal cell differentiation**. (A)** Light microscopy images showing epidermal cell patterning in smooth-skin Numex Garnet and cracked-skin Vezena Slatka fruit at 5 and 50 days after anthesis (daa). **(B)** Epidermal cell density, representing the average number of epidermal cells per cm^2^ of skin surface in 5 daa fruit. Ten skin areas were analyzed for each fruit type, comprising a total of 9218 cells. **(C)** The distribution of the average epidermal cell perimeter in 5 daa fruit. **(D)** Epidermal cell density, representing the average number of epidermal cells per cm^2^ of skin surface, in 50 daa fruit. Fourteen skin areas were analyzed for each fruit type, comprising a total of 1307 cells. **(E)** The distribution of the average epidermal cell perimeter in 50 daa fruit. The dots in all graphs represent individual skin areas. **(F)** Transcript expression levels of key genes involved in epidermal cell patterning biosynthesis in skin tissues of 50 daa fruit from both types. Gene expression levels were measured by quantitative real-time PCR (qRT-PCR) where y-axis represents the normalized expression of each gene following normalization to the two *Capsicum annuum* reference genes *Polyubiquitin-like* (CA00g79660) and *UBI-3* (Capana04g002411). Full gene names appear in the main text and in Materials and Methods. The data in (F) represent means ± SE of three biological replicates (generated from a pool of skin tissue from four different fruit). The significance (B), (C), (D), (E) and (F) was calculated according to a *t*-test of *p*-values: ^*^p < 0.05; ^**^p < 0.01; ^***^p < 0.001. n.s. = non-significant. Scale bar in (A) = 300 μm.

Previous studies have suggested that lower epidermal cell density in fruit skin during its development and enlargement might weaken these cells, resulting in higher susceptibility to rupturing forces [25, 40]. In light of this premise, we sought to evaluate the density of fruit skin epidermal cells and their average perimeter in both cultivars at 5 daa, when cracking is still absent in the skin of Vezena Slatka fruit, and at 50 daa mature fruit (Fig. 3A). Light microscopy revealed that at 5 daa and 50 daa, Numex Garnet fruit skin was composed of a much higher number of epidermal cells with relatively small perimeters compared to Vezena Slatka fruit skin which featured epidermal cells with much larger perimeter (Fig. 3A). In both developmental stages, epidermal cells in the skin of Numex Garnet fruit seem to have relatively similar longitudinal and tangential widths. In the case of Vezena Slatka fruit, however, we found a pattern of epidermal cells with isodiametric and non-isodiametric shape, suggesting a less organized distribution of cells in the skin of these fruit (Fig. 3A).

**Figure 4:**
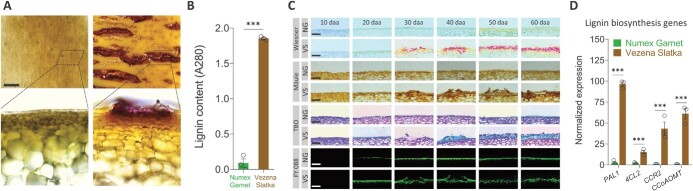
Cracking of the Vezena Slatka fruit skin is accompanied by a spatial accumulation of lignin-like polyphenolic compounds in cracked regions**. (A)** Lignin staining in freshly-cut skin tissues of harvested 60 daa Numex Garnet and Vezena Slatka fruit. Tissues were stained according the Wiesner procedure by Phloroglucinol-hydrochloric acid that yields pink/fuchsia signals indicating the presence of lignin. **(B)** Calorimetric quantification of total lignin contents in skin tissues of harvested 60 daa Numex Garnet and Vezena Slatka fruit. **(C)** Histological sectioning and staining procedures to detect the presence of lignin and suberin in skin tissues of both fruit types during development. Sections were histochemically or fluorescently stained according to the Wiesner procedure with Phloroglucinol reacting with cinnamaldehyde end-groups of lignin; according to the Mäule procedure with Potassium Permanganate and hydrochloric acid that act on the syringyl nucleus of the S-lignin unit to form a methoxycatechol and then to a methoxy-*o*-quinone following the reaction with ammonium hydroxide; with Toluidine Blue O (TBO) that reacts with different chemical components in cells potentially yielding a multi-colored specimen and can be used to indicate cell wall-associated polysaccharides as well as the existence of lignin; and with the Fluorol Yellow 088 (FY 088) fluorescent dye that can react with hydroxycinnamic acids in the fruit skin cuticle in addition to the suberized periderm cells in case formed. **(D)** Transcript expression levels of key genes involved in lignin biosynthesis in skin tissues of 50 daa fruit from both types. Gene expression levels were measured by quantitative real-time PCR (qRT-PCR) where y-axis represents the normalized expression of each gene following normalization to the two *Capsicum annuum* reference genes *Polyubiquitin-like* (CA00g79660) and *UBI-3* (Capana04g002411). Full gene names appear in the main text and in Materials and Methods. The data in (B) and (D) represent means ± SE of three biological replicates (generated from a pool of skin tissue from four different fruit). The significance (B) and (D) was calculated according to a *t*-test of *p*-values: ^*^p < 0.05; ^**^p < 0.01; ^***^p < 0.001. n.s. = non-significant. Scale bars in (A) = 500 μm, and in (C) = 100 μm.

Calculations of epidermal cell densities in 5 daa Numex Garnet fruit showed their skin surface contained an average of 945 epidermal cells/cm^2^ vs. 393 epidermal cells/cm^2^ in Vezena Slatka fruit (Fig. 3B). We attributed this stark difference to the fact that 71% of epidermal cells in the Vezena Slatka fruit had an averaged perimeter >900 μm, whereas almost all epidermal cells belonging to the Numex garnet fruit had significantly smaller perimeters: 15% were between 0–300 μm, 57% between 300–600 μm, and 25% between 600–900 μm (Fig. 3C). Relatively similar trends were detected in 50 daa fruit, where the Numex Garnet fruit skin surface featured a significantly higher epidermal cell density compared to that of Vezena Slatka fruit: an average of 183 vs. 109 epidermal cells/cm^2^, respectively (Fig. 3D). This, too, is the result of the larger-perimeter epidermal cells found in the Vezena Slatka fruit: 10% had an average perimeter of 1000–2000 μm, 21% an average area of 2000–3000 μm, but 42% had an average area > 4000 μm (Fig. 3E). In contrast, 29% of the Numex Garnet fruit surface had epidermal cells with an average perimeter of 1000–2000 μm, 37% with 2000–3000 μm, 18% with 3000–4000 μm, and only 15% with an average perimeter >4000 μm (Fig. 3E). Altogether, these findings imply a fundamental difference in epidermal cell differentiation between the skin of Numex Garnet and Vezena Slatka fruit and suggest that lower epidermal cell densities and large-perimeter cells are possible contributors to fruit skin cracking.

Previous studies were able to identify a subset of genes with possible roles in epidermal cell differentiation. In light of our microscopic observations and calculations of epidermal cell densities and perimeters in the skin of both fruit, we were interested to evaluate the expression of putative orthologous pepper genes with possible roles in epidermal cell differentiation via qRT-PCR. Here, clear differences were detected in the skin of the two fruit, where the majority of examined genes displayed higher expression in Vezena Slatka fruit compared to Numex Garnet. For example, *EXPANSIN 4* (*EXPA4*), which was only recently implicated in pepper fruit skin cracking [41], displayed 5.8-fold higher expression in Vezena Slatka fruit skin (Fig. 3F). The putative orthologs of *ANTHOCYANINLESS 2* (*ANL2*) and *GALBRA 2* (*GL2*), both encoding HD-GLABRA2 group proteins affecting the organization and identity of epidermal and sub-epidermal cells [42–45], displayed ~6-fold higher expressions in Vezena Slatka fruit skin (Fig. 3F). Also, the pepper orthologs of two genes suggested to play a role in epidermal reticulation in tomato fruit *EPIDERMAL RETICULATION* (*ER*) [46] exhibited 4.7- and 7.8-fold higher expression in Vezena Slatka fruit (Fig. 3F). Finally, as to the SHINE clade of transcription factors, which were shown to co-regulate cuticle formation and epidermal cell patterning [39, 47], while the expression of *SHINE 1* (*SHN1*) and *SHN3* pepper orthologs was similar in the skin of both fruit, *SHN2*’s expression was 5.4-fold higher in Vezena Slatka fruit (Fig. 3F). Only recently, the tomato *SHN2* gene was shown to participate in the synthesis of the cuticle along with epidermal patterning formation in the fruit skin [48].

### Cracking of the Vezena Slatka fruit skin is accompanied by a spatial accumulation of lignin-like polyphenolic compounds in cracked regions

Thus far, we gained several lines of evidence that cracking of the Vezena Slatka fruit skin is associated with a thicker cuticular membrane throughout fruit development apparently due to enhanced synthesis of cutin monomers, in addition to lower density of epidermal cells with of large perimeters. Various species of fleshy fruit are capable of establishing a wound-periderm below the cracked skin that is often built from a mass of condensed cells, which separates the cells of the inner pericarp and the upper damaged epidermal cells. It was shown that the cells that build this specialized tissue often accumulate polyphenolic compounds that originate from the biosynthetic pathways of the suberin and lignin aromatic polymers.

To assess the possibility that cracking of Vezena Slatka fruit skin is associated with the buildup of polyphenolic compounds, we initially harvested fresh mature 60 daa (when cracks are the most severe in Vezena Slatka fruit but completely absent in Numex Garnet) fruit and stained them according to the Wiesner procedure that makes use of Phloroglucinol giving pink/fuchsia signals once it reacts with cinnamaldehyde end-groups of lignin, and its intensity often correlates with the level of lignification [49, 50]. Strong signals were spatially confined in the cracked skin of Vezena Slatka fruit, but were completely not detected in Numex Garnet fruit (Fig. 4A). We further extracted the total lignin contents from the skin of these fruit and quantified it calorimetrically, expectedly detecting high lignin contents in Vezena Slatka fruit with virtually no lignin in Numex Garnet fruit (Fig. 4B). Together, these observations suggested that the buildup of lignin is associated with skin cracking in Vezena Slatka fruit.

To strengthen this premise, we comparatively stained histological skin sections from both fruit types along development with Phloroglucinol, in addition to Potassium Permanganate that can specifically react with the S-lignin unit [51]. Phloroglucinol staining demonstrated a stark difference between both fruit where no signs of staining appeared in skin samples of Numex Garnet fruit, while those of Vezena Slatka displayed increasing signs of lignin staining from the appearance of cracks at 30 daa onwards (Fig. 4C). In principal, relatively similar signals were detected as observed in sections stained with Phloroglucinol, suggesting that the lignin accumulated in the cracked skin region of Vezena Slatka fruit is mostly made of the S unit-type components (Fig. 4C).

Next, we used the polychromatic Toluidine Blue O (TBO) that reacts with different chemical components in cells potentially yielding a multi-colored specimen. It was previously shown that TBO can be used to indicate cell wall-associated polysaccharides as well as the existence of lignin [52]. In our case, the walls of epidermal and sub-epidermal cells were stained with a pinkish color pointing at carboxylated polysaccharides that are part of these cell walls (Fig. 4C). Moreover, the cuticle layer deposited above the epidermal cells of Numex Garnet fruit appeared in bright blue suggesting for polyphenolic substances, in agreement with our GC–MS results that indicated the existence of hydroxycinnamic acids in isolated skin cuticles (Fig. 4C). At 20 daa, the cuticle of Vezena Slatka fruit seemed much thicker compared to this of Numex Garnet corroborating our cuticle thickness measurements and cutin monomer profiling. Finally, dissociated cuticle of Vezena Slatka fruit skin from 30 daa onwards was clearly stained with bright blue indicating large amounts of lignin-related polyphenolic compounds (Fig. 4C).

Lastly, we stained skin tissues with the fluorescent Fluorol Yellow 088 (FY 088) dye previously shown to interact with hydroxycinnamic components in the fruit skin cuticle, in addition to the suberized periderm cells in case formed in fleshy fruit such as reticulated melon [13], apple [53, 54], mango [55], and cucumber [11, 14]. Herein, the non-cracked skin of Numex Garnet displayed an intact cuticle throughout fruit development as detected both in Sudan IV (Fig. 2A) and TBO staining (Fig. 4C). In Vezena Slatka fruit, intact cuticle was noticed at 20 daa in which from 30 daa onwards underwent cracking and dissociation (Fig. 4C). Yet, the most valuable observation was that no periderm cells were fluoresced following FY staining indicating that cracking of the Vezena Slatka fruit is apparently not associated with the synthesis of suberin monomers (Fig. 4C). This assumption is supported by our histological investigations that did not point at the formation of a typical periderm cells underneath the cracked skin of Vezena Slatka fruit; but also by GC–MS profiling of skin samples for polymer composition that indicated the existence of C16 and C18 fatty acid derivatives associated with the cutin in Vezena Slatka fruit skin, but not of typical suberin building blocks like fatty acids, fatty alcohols, ω-hydroxyacids and dihydroxyacids with chain lengths >C20.

Thus far, our histological studies and staining procedures provided solid evidence for the spatial accumulation of lignin in the regions of cracks in Vezena Slatka fruit skin. We therefore assumed that the buildup of lignin in the skin of these fruit will be accompanied by induced expression of genes operating in the lignin biosynthesis pathway. We isolated the putative orthologs of four key lignin genes: *PHENYLALANINE AMMONIA LYASE 1* (*PAL1*) that catalyzes the first step in the phenylpropanoid metabolism in which ammonia is being removed from phenylalanine to produce *trans*-cinnamic acid [56]; *4-COUMARATE:CoA LIGASE 2* (*4CL2*) that forms *p*-coumaroyl-CoA from *p*-coumaric acid in an ATP-dependent reaction [57]; *CINNAMOYL-CoA REDUCTASE 2* (*CCR2*) that catalyzes the synthesis of hydroxycinnamaldehydes from the hydroxycinnamoyl-CoA thioesters [58]; and *CAFFEOYL-CoA O-METHYLTRANSFERASE* (*CCoAOMT*) that catalyzed the first methyl transfer reaction in phenylpropanoid metabolism to yield feruloyl CoA from caffeoyl CoA [59]. As expected, gene expression analyses via qRT-PCR indicated 97-, 15-, 43- and 61-fold higher expressions of *PAL1*, *4CL2*, *CCR2* and *CCoAOMT*, respectively, in skin of Vezena Slatka mature 60 daa fruit compared to Numex Garnet (Fig. 4D).

### The effects of skin cracking on pepper fruit response during post-harvest storage

**Figure 5:**
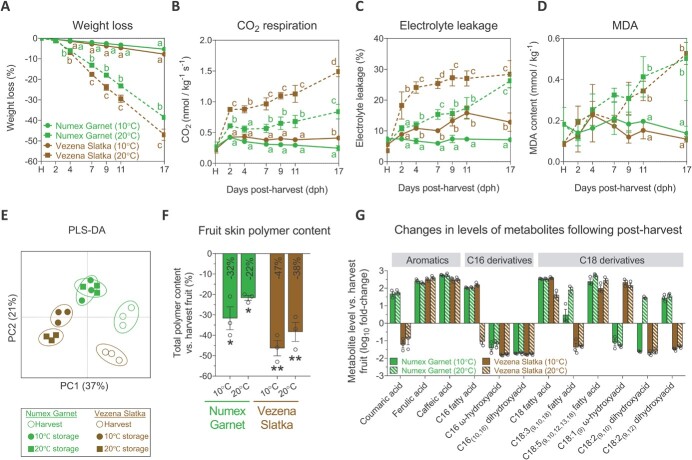
The effects of skin cracking on pepper fruit features during post-harvest storage**.** The **(A)** weight loss; **(B)** CO_2_ respiration levels; **(C)** electrolyte leakage percentages; and **(D)** malondialdehyde (MDA) content in harvested and stored Numex Garnet and Vezena Slatka pepper fruit. The data in (A) represent the means ± SE of nine different fruit. **(E)** Partial Least Squares-Discriminant Analysis (PLS-DA) displaying the spatial distribution of skin samples of Numex Garnet and Vezena Slatka harvested and stored fruit based on their GC–MS polymer profiles. The variances explained by each of the first two components (PC1 and PC2) appear in parentheses. **(F-G)** The changes in (F) the total polymer content and (G) the levels of individual cutin metabolites in the skin of Numex Garnet and Vezena Slatka stored fruit at 10°C and 20°C vs. their levels at harvest. The 0% baseline represents the relative polymer content/metabolite level of harvested fruit in both fruit types. The y-axis in (F) represents the total polymer content (%), and in (G) the log_10_-fold change in metabolite level in stored fruit under the different storage temperatures vs. harvest fruit. The data in (B), (C), (D), (E), (F) and (G) represent means ± SE of three biological replicates (each generated from a pool of at least three different fruit). The significance in (A-D) was calculated according to an ANOVA of *p*-value <0.05. Only significant dph are mentioned by small letters. The significance in (F) was calculated according to a *t*-test of *p*-values: ^*^p < 0.05; ^**^p < 0.01; ^***^p < 0.001.

Recent accumulative evidence highlights the fruit cuticle as an important modulator of post-harvest processes [60]. Thus, we were interested to assess whether cracking of Vezena Slatka fruit skin affects important traits during post-harvest. To this end, we conducted a comparative experiment where we stored 40 daa fruit from both cultivars under 10°C or 20°C for a period of 17 days. During the experiment, we monitored the rates of fruit weight loss, CO_2_ respiration, electrolyte leakage and malondialdehyde (MDA) content at harvest and 2, 4, 7, 9, 11 and 17 days post-harvest (dph) (Fig. 5). In addition, we addressed the effects of the different post-harvest temperatures on fruit skin polymer chemistry by comparing the profiles obtained for skin samples at harvest to those of fruit stored for 17 days.

Over the course of the storage period, the fruit skin of both cultivars stored at 20°C started turning red at 9 dph, suggesting post-harvest ripening processes. This, however, was not the case with fruit stored at 10°C; their skin remained green even after 17 dph (Supplementary Fig. S2). As to the rates of weight loss, Numex Garnet and Vezena Slatka fruit stored at 10°C lost only 5% and 8% of their weight compared to their weight at harvest, respectively (Fig. 5A). This trend intensified under 20°C storage conditions, culminating in an overall weight loss of 39% and 47%, respectively, at 17 dph (Fig. 5A).

Fruit respiration rates, evaluated by measuring CO_2_ emission, of the stored cultivars under 10°C conditions were similar to their harvested fruit counterparts (Fig. 5B). However, under 20°C, both fruit types displayed increased emission of CO_2_, with Vezena Slatka fruit emitting significantly higher levels compared to Numex Garnet fruit throughout the storage period (1.49 and 0.84 nmol/kg^−1^ S^−1^, respectively, at 17 dph) (Fig. 5B).

Stabilizing their membrane integrity allows fruit to sustain their fitness and viability during post-harvest storage. Damage to the membrane integrity during storage is often accompanied by higher ion leakage and the buildup of MDA, which is the product of lipid peroxidation processes. We detected a gradual increase in the percentage of electrolyte leakage in both cultivar fruit under 10°C, with Vezena Slatka fruit exhibiting significantly higher percentages at 2, 4, 7, 9 and 11 dph compared to Numex Garnet fruit (Fig. 5C). Here, too, the trend was more pronounced under 20°C storage conditions: Even though fruit of both cultivars reached a similar value of ~28% electrolyte leakage after 17 dph, the incline detected in Vezena Slatka fruit was much greater (Fig. 5C). In line, the MDA contents were higher in fruit stored under 20°C compared to fruit stored under 10°C, particularly from 7 dph to 17 dph, when Numex Garnet and Vezena Slatka fruit accumulated a relatively similar amount of MDA content (0.51 and 0.53 nmol/kg^−1^ at 17 dph, respectively) (Fig. 5D).

### Chemical rearrangements in fruit skin cutin monomers during post-harvest storage

Next, we evaluated the changes in skin cutin content and composition following post-harvest. To this end, we compared the polymer composition of skin samples isolated from 50 daa harvested fruit to those isolated from 50 daa fruit being stored at 10°C and 20°C for 17 days. We first plotted the samples of harvested and stored fruit according to their skin polymer profiles onto a partial least square-discriminant analysis (PLS-DA) in order to detected general trends of chemical changes in the skin during post-harvest storage. The analysis delineated two general trends: (i) post-harvest storage, both under 10°C and 20°C, altered the fruit skin’s cutin profile compared to at harvest; (ii) each storage temperature affected the smooth and cracked fruit in a different manner, as skin samples from Numex Garnet fruit stored at 10°C and 20°C were grouped together, while those from the Vezena Slatka fruit formed a separate group (Fig. 5E). Our calculation of the two fruits’ total skin polymer content under both storage conditions revealed that post-harvest storage significantly reduced the cutin content in all the studied fruit. However, while Numex Garnet fruit stored at 10°C and 20°C exhibited a 32% and 22% reduction in their cutin content, respectively, that of the Vezena Slatka fruit stored under these temperatures was more pronounced, 47% and 38%, respectively (Fig. 5F).

By monitoring the level of each cutin metabolite, we were able to determine that the reductions were primarily due to significantly lower levels of C16 $ \omega $-hydroxyacid and C16_(10,16)_ dihydroxyacid, the two most abundant cutin monomers (Fig. 5G). Interestingly, the large reductions detected in stored Vezena Slatka fruit were due to significant lower levels of other metabolites such as coumaric acid, C18:2_(9,10)_, C18:2_(9,12)_ dihydroxyacids, and C18:3_(9,10,12)_ fatty acid, all of which displayed higher abundances in stored Numex Garnet fruit (Fig. 5G). Increased levels of ferulic and caffeic acids, C18 and C18:5_(9,10,12,13,18)_ fatty acids were detected in both stored fruit types under the two temperatures (Fig. 5G). C18:1_(9)_$ \omega $-hydroxyacid was the only metabolite that was significantly depleted in Numext Garnet fruit while showcasing high levels in Vezena Slatka stored fruit (Fig. 5G).

To conclude, our post-harvest assays suggest that the cracking of the fruit skin in pepper leads to higher rates of weight loss, CO_2_ respiration, ion leakage and MDA accumulation, particularly under higher storage temperatures. Moreover, post-harvest storage results in reduced fruit skin cutin content, mostly as a consequence of a reduction in the levels of abundant cutin building blocks, a trend that seems to be more pronounced in cracked-skin pepper fruit.

## Discussion

### Skin cracking in pepper fruit is concomitant with the buildup of lignin but does not accompany a typical wound-periderm tissue made of suberin

Skin cracking occurs in many fruit varieties, including apple, sweet cherry, grape, pomegranate, persimmon, lychee, and tomato [61, 62]. This skin failure reduces the quality of pre- and post-harvest fruit, and has therefore generated greater interest in recent years. Prior studies have revealed the molecular and biochemical mechanisms that partake in the formation of wound-periderm tissues that complement skin cracking in tomato [63], kiwi [64], apple [65], melon [13], sand pear [66], and cucumber [14]. These investigated also demonstrated the spatial localization of both suberin and lignin polymers in the peridermal cell layer underneath the cracked skin. In contrast to these studies, we did not detect a specialized peridermal cell layer nor the accumulation of suberin in Vezena Slatka fruit. Rather, we observed the buildup of lignin that coincided with the appearance of cracks. This finding was further supported by our metabolite profiling assays, which indicated much higher levels of hydroxycinnamic acids (i.e. coumaric, ferulic and caffeic acids), apparently originating from the lignin polymer in the cracked-skin of Vezena Slatka fruit. At the same time, we could not detect any of the VLCFA derivatives (typically with chain-length > C20) that build the suberin polymer. In other fruit species, the formation of periderm tissue and the accumulation of suberin usually occur at the expense of cutin biosynthesis, leading to lower levels of cutin components upon the appearance of cracking [14, 65, 67]. In our investigated chili-type pepper fruit varieties, we did not see any reduction in cutin’s predominant monomers in Vezena Slatka fruit compared to Numex Garnet, further strengthening the conclusion that no periderm tissue was formed in Vezena Slatka fruit. Indeed, it was previously suggested that not all fruit species are capable of establishing a specialized periderm tissue. In such cases, it was proposed that the rigidification of cell walls underneath the cracks via the deposition of lignin facilitates a protective barrier that assists the fruit to limit the consequent damage arising from skin failure [12].

### Cuticle thickness and epidermal cell density are two critical factors determining fruit skin susceptibility to cracking in chili-type pepper fruit

Our findings provide solid evidence that cuticle thickness and epidermal cell density are apparently two critical factors that possibly account for Vezena Slatka fruit heightened susceptibility to skin cracking during early stages of development. At 20 daa, when there were still no cracks on the skin surface, Vezena Slatka fruit already accumulated significantly higher levels of four key C16-C18 cutin components (i.e. C16_(10,16)_ and C18:2_(9,10)_ dihydroxyacids, and C18:3_(9,10,18)_ and C18:5_(9,10,12,13,18)_ fatty acids) compared to Numex Garnet fruit. This might be one of the main reasons that we measured a significantly thicker skin cuticle at early developmental stages of Vezena Slatka fruit. This concept was partially explained by gene-expression assays showing an induced expression of several key genes involved in cutin biosynthesis. Hence, our data suggest a negative correlation between cuticle thickness and cracking susceptibility in chili-type pepper fruit. While mining the literature, we found inconsistencies regarding the contribution of cuticle thickness, among other cuticle properties, to cracking resistance. For instance, a positive correlation between the two parameters was noted in tomato fruit [68]. Similarly, low cuticle deposition rates during the development of sweet cherry and mango fruit increased the elastic strain and weakened the cuticle, thereby caused skin failure [55, 69]. Yet, cuticle thickness was not associated with cracking resistance in grape berry splitting and apple fruit russeting [67, 70–73]. Thus, it is apparent that there is no clear correlation between cuticle thickness and skin cracking susceptibility, and likely other traits, such as cuticle structure, density, assembly and composition, are determining factors in these processes.

The second factor that contributes to Vezena Slatka fruit’s heightened susceptibility to skin cracking during early stages of development is lower epidermal cell density. The perimeter of epidermal cells that formed the skin of the Vezena Slatka fruit was significantly larger compared to those of the Numex Garnet fruit at both early and late developmental stages. Moreover, the epidermal cells in Numex Garnet fruit skin displayed relatively similar longitudinal and tangential widths, while those of Vezena Slatka fruit exhibited a more disorganized pattern composed of epidermal cells with isodiametric and non-isodiametric shape. This, in turn, resulted in lower epidermal cell densities per the same unit of surface area in Vezena Slatka fruit. In other words, our data suggest that higher densities of epidermal cells in Numex Garnet fruit skin make it more resilient to cracking. This concept is in line with previous studies demonstrating that an increase in epidermal cell density accompanies deceased skin cracking and russeting in apple fruit [71, 72, 74, 75], cranberries [76], sweet cherry [77] and tomato [78]. Markedly, Vezena Slatka fruit skin exhibited a significant higher expression of genes with demonstrated roles in both cuticle assembly and epidermal cell patterning, i.e. *EXPA4*, *ANL2*, *GL2*, *ER* and *SHN2*. Our data, therefore, provide an additional line of evidence that the gene networks regulating the formation and differentiation of the epidermis in the skin of fleshy fruit are predominant factors that assert skin integrity and resistance to cracking. At the same time, we cannot rule out that the differences in cuticle biosynthesis and deposition at early developmental stages of the two fruit varieties did not affect epidermal cell patterning. Indeed, it has been postulated that cuticle disruption or impairment can alter cell signaling, thus affecting epidermal cell differentiation [79].

### Skin cracking affects fruit features during post-harvest storage

Post-harvest storage is a critical stage in agricultural crops. Multiple morpho-chemical processes that occur during storage dramatically influence fruit quality and traits. The loss of water via transpiration and respiration are the major factors leading to weight losses of fleshy fruit [80]. Therefore, water loss and CO_2_ respiration rates are among the most common parameters measured during post-harvest storage experiments. Our study indicates that storing pepper fruit at 20°C considerably accelerates the rates of water loss and CO_2_ respiration in both fruit types, compared to fruit stored at 10°C. The warmer storage conditions also accelerated post-harvest ripening processes, with fruit turning red already at 9 dph under 20°C, while fruit stored under 10°C remained stiff and green even after 17 dph. Yet, while both fruit displayed similar trends of water loss and CO_2_ respiration during storage, the rates measured in Vezena Slatka fruit were significantly higher compared to Numex Garnet, particularly at 20°C. Our data is in line with a previous report that Vezena Slatka fruit loses water at faster rates due to the presence of cracks on top of their skin [27]. Another study showed that water vapor permeance via the skin is three times greater in cracked-skin Jalapeno chili pepper fruit compared to non-cracked fruit [81]. Interestingly, we recently showed that cracking in the skin of the Sikkim cucumber fruit (*Cucumis sativus* var. *sikkimensis*) actually reduces the rates of water loss and respiration during storage compared to those of the common cucumber fruit (*Cucumis sativus*) [11]. However, unlike Vezena Slatka fruit, the Sikkim cucumber produces a typical wound-periderm tissue made of suberin and lignin polymers that apparently acts as an efficient hydrophobic barrier, lessening transpiration and respiration through the skin.

Maintaining the membrane integrity of skin cells is a critical factor during post-harvest storage, which significantly effects fruits’ shelf life. Damages to cell membranes during storage often results in elevated electrolyte leakage rates and the accumulation of MDA [82]. As noted for water loss and respiration, the rates of electrolyte leakage and buildup of MDA in the skin of both pepper fruit were higher when stored at 20°C compared to 10°C. However, under both temperatures, Vezena Slatka fruit displayed significantly higher leakage rates. The most pronounced difference was at 20°C, where 25% of Vezena Slatka fruit skin cell membranes were already damaged at 4 dph. We, therefore, conclude that the severe cracking in the skin of these fruit has detrimental consequences to cell membrane integrity. MDA, however, accumulated in the skin of both fruit at similar levels, suggesting a more comparable effect of storage temperature on the accumulation of MDA.

Overall, our data reveal significant disparate responses of the two pepper fruit varieties during post-harvest storage, a trend that become more pronounced under 20°C storage. The Numex Garnet fruit outperformed Vezena Slatka, highlighting the detrimental outcomes of skin cracking to pepper fruit, marked by higher weight loss and greater damage to cell membranes.

### The effect of post-harvest storage on the fruit skin cuticle

The skin cuticle acts as a key modulator of fruit quality during post-harvest storage [60]. Recent accumulative data suggest that chemical changes in the skin cuticle components (i.e. epicuticular waxes and cutin monomers) occur in the post-harvest fruit of apple, peach, sweet cherry, strawberry, blueberry, pear, tomato, orange, persimmon, mango and zucchini [83]. Our post-harvest assays revealed a reduction in the polymer content in the skin of both pepper fruit types under 10°C and 20°C storage conditions, with those stored at 10°C displaying more substantial reductions. These observations are in line with reports of lowered total cutin content in orange (*Citrus sinenis* L. Osbeck) fruit stored at 4°C and 25°C for 40 days [84], and cucumber (*C. sativus*) fruit stored at 10°C [11]. Our data do not reveal a clear pattern of change in specific cutin monomers and/or biochemical classes that are part of pepper fruit skins’ response to storage. Nevertheless, with respect to differential metabolic changes that might be liked to skin cracking, coumaric acid, C18:1_(9)_$ \omega $-hydroxyacid, C18:2_(9,12)_ dihydroxyacid and C18:3_(9,10,18)_ fatty acid all exhibited opposite accumulation patterns in Vezena Slatka fruit skin compared to Numex Garnet under both storage temperatures. To the best of our knowledge, there are no reports in the literature on the effect of fruit skin cracking on cuticle content and composition following post-harvest storage, apart from our recent report on cucumber fruit skin reticulation [11]. However, in cucumber fruit, skin cracking was associated with a significant accumulation of suberin, a feature not detected in the cracked skin of Vezena Slatka fruit.

In conclusion, the chemical modifications in skin polymers following post-harvest storage detected in the two pepper fruit varieties amalgamate several other lines of evidence highlighting skin polymers as factors that are imperative for fruit quality following post-harvest. At the same time, the limited knowledge on these processes raises the need for additional research into the relationship between fruit cuticle metabolism, skin cracking and post-harvest storage performance.

## Conclusions

Our microscopic assays coupled with gas chromatography-metabolite profiling revealed that skin cracking in Vezena Slatka fruit is concomitant with the spatial accumulation of lignin in cracking regions, but not the formation of a typical wound-periderm tissue made of suberized cells. Moreover, the cuticle of these fruit seem to be much thicker throughout early and late stages of development, with higher accumulation of several C16–18 fatty acid and $ \omega $-hydroxylated derivatives being a reasonable accounting factor. We further found that at early and late developmental stages, Numex Garnet fruit contain higher epidermal cell densities, a consequence of their smaller perimeters compared to those found in Vezena Slatka fruit. This finding further implies that the cracking in Vezena Slatka chili-type fruit is affected by the lower density of epidermal cells with much larger perimeters and the disorganized pattern of epidermal cells with isodiametric and non-isodiametric shape. Lastly, we show that during post-harvest storage of both fruit varieties at 20°C, skin cracking leads to higher rates of weight loss, CO_2_ emission and ion leakage, but not when stored at 10°C. Altogether, our findings provide valuable insight concerning the factors contributing to skin cracking in chili-type pepper fruit varieties and the effects of skin cracking on pre- and post-harvest fruit quality.

## Materials and methods

### Plant material and growth conditions

In the current study, we used two pepper (*Capsicum annuum* L.) cultivars. The first is “Numex Garnet”, a paprika-type fruit released by the New Mexico Agricultural Experiment Station [26], and the second is “Vezena Slatka” - a chili-type fruit obtained from a collection belonging to the USDA-GRIN (Germplasm Resources Information Network) [27]. Pepper plants from the two cultivars were grown in a greenhouse with controlled environmental conditions in 7 L pots, and periodically supplemented with a 20:20:20 N:P:K fertilizer. Fruit were collected for further analyses at 10, 20, 30, 40, 50, and 60 days after anthesis (daa). Skin tissues from these fruit were immersed in freshly-prepared histology or electron microscopy fixation buffers, de-lipidated for further polymer profiling analyses via GC–MS, prepared for lignin quantification experiment, stored at −80°C for RNA extraction and qRT-PCR assays, or stored under different post-harvest conditions for storage experiments.

### Scanning electron microscopy (SEM)

Skin samples were dissected from 10, 20, 30, 40, 50, and 60 days after anthesis (daa) Numex Garnet and Vezena Slatka fruit, fixed in 100% methanol for 10 min, transferred into clean 100% ethanol for 30 min, and eventually dehydrated in 100% absolute ethanol overnight. Dehydrated fixed samples were mounted on SEM holders and underwent full dehydration according to the critical point dehydration protocol (CPD) using a Quorum K850 critical point dry system and CO_2_ maintaining the original tissue structure [85]. Prior SEM observations using a JEOL 7800 FEG SEM microscope at 5 to 10 kV, samples were coated with a 2 nm layer of Au/Pd particles using a Quorum Q150T ES turbo-molecular pumped sputter.

### Histological sectioning and staining procedures

In order to evaluate the anatomy and structure of the skin tissues of both fruit, skin samples were dissected from 10, 20, 30, 40, 50, and 60 daa fruit and immediately fixed in freshly-prepared FAA solution (formaldehyde:acetic acid). Fixed samples were dehydrated in a graded ethanol series and finally infiltrated with paraffin as described by [13]. Twelve-mm histological cross-sections of skin tissues were generated using a Rhenium Leica microtome and eventually mounted on glass slides.

The cuticle was stained by a freshly-prepared solution of Sudan IV. Stock solution of 0.1% [w/v] was prepared by adding 100 mg of Sudan IV powder (BDH Chemicals) to 100 ml of isopropyl alcohol, which was then diluted at a ratio of 3:2 with distilled water. Following an incubation of 30 min at room temperature the stock solution was filtered to remove precipitants. Sections were stained for 20 min at room temperature, and subsequently rinsed with 50% isopropyl alcohol and distilled water. The presence of lignin was evaluated by staining sections with a freshly-prepared solution of Phloroglucinol:hydrochloric acid. Stock solution of 3% [w/v] was prepared by adding 300 mg of Phloroglucinol powder (Sigma-Aldrich) to 100 ml of absolute alcohol, which was then diluted at a ratio of 2:1 with 12 N hydrochloric acid. Sections were stained for 30 min at room temperature. Identifying signals of syringyl nucleus of the S-lignin unit were achieved by staining sections with a freshly-prepared solution of Potassium Permanganate. Staining solution of 0.5% [w/v] was prepared by adding 400 mg of Potassium Permanganate powder (Fisher Chemical) to 80 ml of distilled water. Sections were stained for 6 min at room temperature at dark, and subsequently rinsed with distilled water, 3.7% hydrochloric acid, and then transferred to 14.8 M Ammonium Hydroxide solution (Sigma-Aldrich). The presence of cell wall-associated polysaccharides as well as lignin were detected by staining section with a freshly-prepared solution of Toluidine Blue O. Stock solution of 0.01% [w/v] was prepared by adding 10 mg of Toluidine Blue O powder (Sigma-Aldrich) to 100 ml distilled water. Sections were stained for 1 min at room temperature, and subsequently rinsed with distilled water. Lastly, the presence of hydroxycinnamic acids in the cuticle layer and suberin (in case a suberized wound-periderm is formed) were evaluated by staining sections with a freshly-prepared solution of Fluorol Yellow 088. Stock solution was prepared by adding 10 mg of Fluorol Yellow 088 powder (Santa Cruz Biotechnology) to 100 ml of lactic acid. Sections were stained for 1 h at 70°C, and subsequently rinsed with distilled water. Images following all staining procedures were captured using a Nikon H600L Eclipse Ni light microscope equipped with a digital Nikon DS-Ri2 camera, using bright-field or GFP filters.

### Metabolite profiling of fruit skin polymers

Cutin and suberin monomers from Numex Garnet and Vezena Slatka fruit skin discs were dissected from 10, 20, 30, 40, 50 and 60 daa fruit. All procedures of sample de-lipidation, trans-esterification and derivatization, as well as and GC–MS apparatus and running parameters, are exactly as those recently described in-detail [11, 14, 28].

### Calorimetric measurements of total lignin

Calorimetric quantification of total lignin was performed as previously described [86]. Skin samples from Numex Garnet and Vezena Slatka 60 daa fruit were dissected and ground into fine powder in liquid nitrogen. Two hundred mg were immersed in 1 ml of 50% ethanol for 3 hours at 80°C, following by the addition of 1 ml of methanol for an additional 1 hour at the same temperature. Following centrifugation and removal of the supernatant, the residual tissue was dried to powder at 60°C overnight. Seventy five mg of dry powder were extracted in 5 ml of 25% (w/w) acetyl bromide in acetic acid for 30 min at 70°C, following the addition of 0.2 ml of 70% perchloric acid for additional 30 min at the same temperature. Treated samples were allowed to cool down, underwent centrifugation at 3000 x *g* for 15 min and the supernatant was transferred into new 50 ml tubes. Five ml of 2 M NaOH were added to each tube and the total mixture volume was filled up to 25 ml with the supplement of glacial acetic, followed by vigorous vortexing. Lastly, 1 ml of the mixture was measured for absorbance at 280 nm under a UV Gene Quant pro spectrophotometer.

### Measurements of weight loss rates, CO_2_ concentration, malondialdehyde (MDA) content and electrolyte leakage rate during post-harvest storage

Weight loss rates, CO_2_ concentrations, malondialdehyde (MDA) contents and electrolyte leakage rates in skin of Numex Garnet and Vezena Slatka fruit at harvest and during post-harvest experiment [measured at 2, 4, 7, 9, 11 and 17 days post-harvest (dph) and under storage at 10°C or 20°C] were performed as described recently by [11].

### Gene expression analyses via quantitative real-time PCR

Total RNA was extracted from mature pepper fruit using the InviTrap® Spin Plant RNA Mini Kit (INVITEK Molecular) according to the protocols of the manufacturer. Removal of excessive genomic DNA was performed by treating RNA samples with QIAGEN RNase-free DNase. Final RNA concentrations and of purity ratios were measured using a standard NanoDrop. Preparation of complementary DNA (cDNA) samples for qRT-PCR gene expression assays was performed using the Thermo Scientific RevertAid First-Strand cDNA Synthesis Kit according to the protocols of the manufacturer. We analyzed the transcript expression levels of key genes involved in cutin biosynthesis (Fig. 2), epidermal cell patterning (Fig. 3) and lignin biosynthesis (Fig. 4), and used the two *Capsicum annuum* endogenous genes *Polyubiquitin-like* (CA00g79660) and *UBI-3* (Capana04g002411) as normalizing genes as described by [87]. The Fast SYBR™ Green Master Mix was used to amplify cDNA samples that were analyzed by a qRT-PCR machine (Applied Biosystems by Thermo Fisher Scientific) according to the thermal cycle program recently described by [88].

### Statistical analyses

The GraphPad Prism v8.0.1 (https://www.graphpad.com/) software was used to generate graphs and perform adequate statistical analyses. The significance was calculated according to the student *t*-test of *p*-values: *p < 0.05; **p < 0.01; ***p < 0.001 (Figs. 2, 3 and 4), or the one-way ANOVA test of *p*-value *<*0.05 (Fig. 5) determined using the Holm-Sidak method. The number of biological replicates is mentioned in the legend of each figure. Partial least square-discriminant analysis (PLS-DA) of fruit skin polymer compositions at harvest and stored fruit was performed using MetaboAnalyst v5.0, (http://metaboanalyst.ca/) [89, 90].

## Acknowledgments

The authors would like to thank Ilan Paran from the Department of Vegetable and Field Crops, Institute of Plant Science, Agricultural Research Organization (ARO), Volcani Center, for providing seeds of Numex Garnet and Vezena Slatka pepper fruit. We also acknowledge Amnon Lers Daniel Chalupowicz from the Department of Post-harvest Science, Institute of Post-harvest and Food Sciences, Agricultural Research Organization (ARO), Volcani Center, for their guidance during the post-harvest experiments. The authors would also like to thank Ms. Natalie Page for proofreading the manuscript.

## Author contributions

O.M. executed all the experiments in this project; G.N., S.S., G.C.A. and E.M. assisted in epidermal cell counting experiments and microscopy; E.K. assisted in the cultivation of pepper and greenhouse maintenance; E.Z. assisted in electron microscopy experiments; O.M. and H.C. conceptualized and designed the project, and wrote the manuscript. All authors approved the final version of the manuscript.

## Data availability

The authors declare that all the data supporting the findings of this study are available within the manuscript and its supplemental information files.

## Conflict of interest

The authors declare they have no competing financial interests or personal relationships that could have influenced the work reported in this manuscript.

## Supplementary Data

Supplementary data is available at *Horticulture Research* online.

## Supplementary Material

Web_Material_uhad036
