# Supplementary material for: Microscopic and metabolic investigations disclose the factors that lead to skin cracking in chili-type pepper fruit varieties
Source: Hortic Res. 2023 Feb 28;10(4):uhad036. doi: 10.1093/hr/uhad036 (PMC10548408; doi:10.1093/hr/uhad036)
Supplement: Web_Material_uhad036 [file web_material_uhad036.pdf]

<u>**Supplementary information**</u>

Microscopic and metabolic investigations disclose the factors that lead to skin cracking in chili-type pepper fruit varieties

Ofir Marinov[1,2], Gal Nomberg[1,2], Sutanni Sarkar[1,2], Gulab Chand Arya[1], Eldad Karavani[1], Einat Zelinger[3], Ekaterina Manasherova[1], Hagai Cohen[1,✉]

[1] Department of Vegetable and Field Crops, Institute of Plant Sciences, Agricultural Research Organization (ARO), Volcani Center, Rishon LeZion 7505101, Israel

[2] Department of Plant Pathology and Microbiology, The Robert H. Smith Faculty of Agriculture, Food and Environment, The Hebrew University of Jerusalem, Rehovot 7610001, Israel

[3] Center for Scientific Imaging (CSI), The Robert H. Smith Faculty of Agriculture, Food and Environment, The Hebrew University of Jerusalem, Rehovot 7610001, Israel

✉Corresponding author e-mail address: hagaic@volcani.agri.gov.il

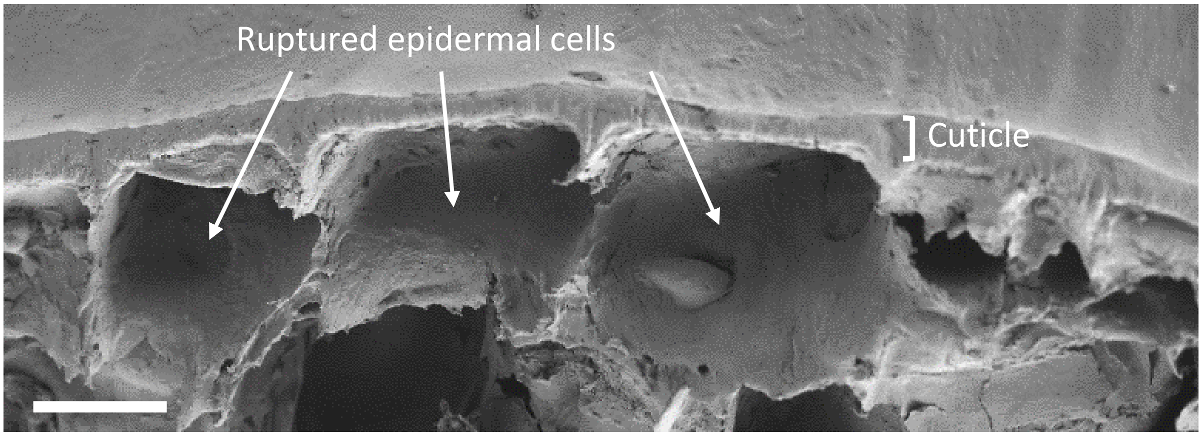

Supplementary Fig. S1. High-resolution SEM images of skin cracking in Vezena Slatka 60 daa fruit. White arrows point at the outermost raptured epidermal cell layer coated by the cuticle that are lifted up in the skin cracking areas. Scale bar = 20 µm.

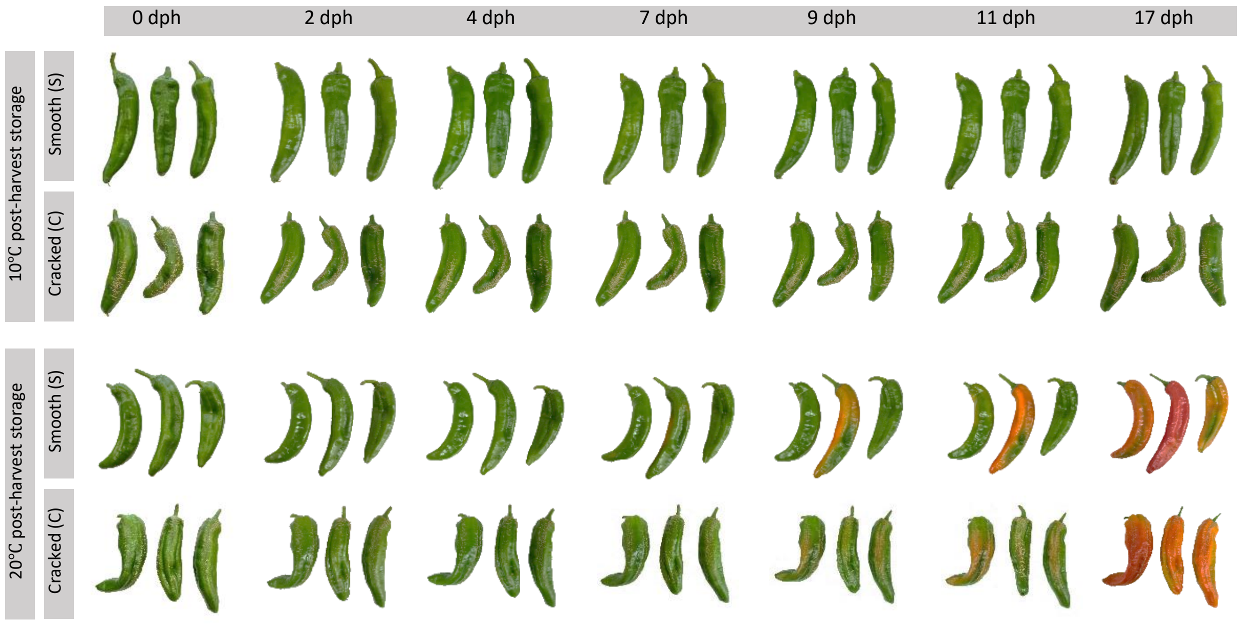

Supplementary Fig. S2. Fruit phenotypes during post-harvest storage. Images of Numex Garnet and Vezena Slatka 40 daa fruit stored under 10℃ or 20℃ at 0, 2, 4, 7, 9, 11, and 17 days post-harvest (dph). Scale bar = 20 µm.

Supplementary Table S1. List of primers used for qRT-PCR assays.

Enzyme name	Enzyme full name	Pepper putative homolog	Primers (5'-3')
LACS2	*LONG-CHAIN ACYL-COA SYNTHETASE 2*	CA08g18140	F TCAAATGCTGGGACGCCGTCAG<br>R ACACCACGGCTTCGAATGGCTG
CD1	*CUTIN DEFICIENT 1*	CA01g08570	F TTGCCCAACGTAGCAGGAACGG<br>R ACATTGCCGCCCAGTTCACTGT
GDSL1	*GDSL-MOTIF ACYLTRANSFERASE 1*	CA02g12870	F TGCCTCTGCAGCTGCTGGAATT<br>R TGCACCACTTGTTGCACTGTGT
DCR	*DEFECTIVE IN CUTICULAR RIDGES*	CA03g18590	F CCGCCATTGCAGTCCCACCTTT<br>R TTGCTGACTTGGCATGCTCGGG
GPAT6	*GLYCEROL3-PHOSPHATE ACYLTRANSFERASE 6*	CA09g08990	F ACCCTGTTGTCACAGCTGTTGCA<br>R CTCTCTTGACAACGCCACGGCT
HTH	*HOTHEAD*	CA01g03530	F TGGGTTGGGAGGGTGGTTTGGT<br>R TCCGACCTCCACAAGTCCGTCG
FDH	*FIDDLEHEAD*	CA11g15140	F GCGCCACCCTCCATCGATTTGG<br>R TTTGCCACACACGATCCCCTCT
CYP77A1	*CYTOCHROME P450 77A1*	CA12g05870	F ATCCTGGGTCCGACAAGGCTGC<br>R AAGCTCTGGATTCGTCGGCCCT
CYP86A8	*CYTOCHROME P450 86A8*	CA08g07320	F GCTCGAAGCCAAGCCGGTTGAT<br>R GGGTGCCAATGTGTGTGGGTCC
EXPA4	*EXPANSIN 4*	CA06g04830	F CGTCGGGTGCCATGTAGGAAGC<br>R GTCACCTGCTCCCGCGACATTG
ANL2	*ANTHOCYANINLESS 2*	CA01g19070	F GCGCCAGTTGATATCCCGGCAA<br>R TCCTGGGCCGTCGGGTACAATT
GL2	*GLABRA 2*	CA03g34750	F ACGCTGCAAGGTAACGTGGGTG<br>R GAGTAGCCATCCAGCGCCTTGC
ER4.1	*EPIDERMAL RETICULATION 4.1*	CA12g18170	F ACGAGTGGGGATACAGCCAACGT<br>R TCCTCCAAGGCGTCTCCACTCC
ER4.2	*EPIDERMAL RETICULATION 4.2*	CA04g23560	F AGCGGATCGGCAGGAGGAGTAC<br>R GCGTCTTCCCCGCAAACCTCTT
SHN1	*SHINE 1*	CA03g31760	F GTGCAGGCACCTGACGACGAAA<br>R TCTTGCGGAGTTTGGCGCTCAG
SHN2	*SHINE 2*	CA06g14570	F TGGGTCTCTGAAATTCGCCATCCA<br>R TGGACCACACATTAGCCTTGCTGC
SHN3	*SHINE 3*	CA03g32280	F GTATCGTGGAGTTCGCCAGCGG<br>R TGCTCTGGCTGCATCCTCTGCT
PAL1	*PHENYLALANINE AMMONIA-LYASE 1*	CA09g02410	F TGGGCGCTAACGGTGAACTTCA<br>R AGGGTAGGTTGAGCTGCAGGGA
4CL	*4-COUMARATE COA LIGASE*	CA06g17350	F GCTCCTGCTGAACTCGAAGCCC<br>R GCCACTGGAACTTCTCCTGCTTGC
CCR2	*CINNAMOYL COA REDUCTASE*	CA03g32090	F TGCTGAGAGCGTGCTTCATCGC<br>R TTGCCCTAGGCCTCGTCTCGTC
CCoAoMT1	*CAFFEOYL COENZYME A 3-O-METHYLTRANSFERASE 1*	CA02g14450	F CCACCTGATGCACCCCTGAGGA<br>R CCCAACGGGAAGCTGGCAGATT
UBI-3	*UBIQUITIN-CONJUGATING PROTEIN 3*	CA06g03040	F CTGTTGACGGACCCAAACCCCG<br>R TGAGTCCAGCTACGAGCAGTGGT
REV05		CA00g79660	F GGACCAGCAAAGGTTGATTT<br>R CAGATGGAGGGTTGATTCCT
